# UV-B–induced forest sterility: Implications of ozone shield failure in Earth’s largest extinction

**DOI:** 10.1126/sciadv.1700618

**Published:** 2018-02-07

**Authors:** Jeffrey P. Benca, Ivo A. P. Duijnstee, Cindy V. Looy

**Affiliations:** 1 Department of Integrative Biology and Museum of Paleontology, University of California, Berkeley, 3060 Valley Life Sciences Building, Berkeley, CA 94720–3140, USA.; 2 University and Jepson Herbaria, University of California, Berkeley, 1001 Valley Life Sciences Building #2465, Berkeley, CA 94720–2465, USA.

## Abstract

Although Siberian Trap volcanism is considered a primary driver of the largest extinction in Earth history, the end-Permian crisis, the relationship between these events remains unclear. However, malformations in fossilized gymnosperm pollen from the extinction interval suggest biological stress coinciding with pulsed forest decline. These grains are hypothesized to have been caused by enhanced ultraviolet-B (UV-B) irradiation from volcanism-induced ozone shield deterioration. We tested this proposed mechanism by observing the effects of inferred end-Permian UV-B regimes on pollen development and reproductive success in living conifers. We find that pollen malformation frequencies increase fivefold under high UV-B intensities. Surprisingly, all trees survived but were sterilized under enhanced UV-B. These results support the hypothesis that heightened UV-B stress could have contributed not only to pollen malformation production but also to deforestation during Permian-Triassic crisis intervals. By reducing the fertility of several widespread gymnosperm lineages, pulsed ozone shield weakening could have induced repeated terrestrial biosphere destabilization and food web collapse without exerting a direct “kill” mechanism on land plants or animals. These findings challenge the paradigm that mass extinctions require kill mechanisms and suggest that modern conifer forests may be considerably more vulnerable to anthropogenic ozone layer depletion than expected.

## Teaser

UV-B regimes modeled for Earth’s largest mass extinction sterilize living conifers.

## INTRODUCTION

**Fig. 1:**
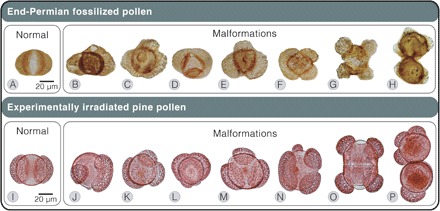
Normal and malformed bisaccate pollen. (**A** to **H**) Fossilized pollen of end-Permian gymnosperms from the Guodikeng Formation, Dalongkou section, North China (A to G) and Nedubrovo, Russia (H). (A) *Alisporites* sp., bisaccate. (B) Bisaccate indet., possibly taeniate with asymmetric sacci. (C) *Klausipollenites* sp., trisaccate. (D to F) *Alisporites* sp., trisaccate. (G) *K. schaubergeri*, tetrasaccate dyad. (H) *K. schaubergeri*, fused pollen including a trisaccate. (**I** to **P**) Pollen of modern pine (*P. mugo* Columnaris) irradiated with modeled end-Permian UV-B regimes. (I) Bisaccate. (J) Trisaccate grain with enlarged saccus. (K) Trisaccate grain with enlarged saccus. (L) Trisaccate grain with fused sacci. (M) Trisaccate grain. (N) Tetrasaccate grain. (O) Tetrasaccate dyad. (P) Fused grains, one trisaccate. See Supplementary Text for details and image credits.

The end-Permian crisis (~251.9 million years ago) (*1*), the largest mass extinction in Earth history, initiated a series of floral destabilization-recovery episodes that continued ~500 thousand years (ky) into the succeeding Triassic Period (*2*, *3*). Massive volcanism associated with the formation of the Siberian Traps igneous province is considered a likely driver behind these events (*4*). Although many Traps-related kill mechanisms have been proposed, the proximal causes of ecosystem-scale disturbance are unclear. Among the few inferred direct clues of biological stress in the fossil record are malformed bisaccate pollen grains (Fig. 1, B to H) (*5*). Bisaccate pollen grains are wind-dispersed grains that normally consist of a central body with two symmetric, bladder-like appendages called sacci. Sacci are inflated regions of the outermost pollen wall that function to increase the buoyancy of pollen grains, enabling their flotation against gravity through downward-facing liquid pollination drops associated with the inverted (upside-down–facing) ovules of several conifer and seed fern groups (*6*, *7*). This pollen form is produced abundantly by certain gymnosperm lineages and has remained a conspicuous element of palynomorph assemblages since the Pennsylvanian (*8*). Bisaccate pollen malformations are likely products of meiotic disruption because they have asymmetric sacci, more or less than two sacci, and are occasionally fused or unseparated (Fig. 1, B to H) (*5*).

In end-Permian fossil pollen assemblages studied for abnormalities, 3 to 6% of two bisaccate pollen types, *Klausipollenites schaubergeri* and *Alisporites* sp., appear malformed in coeval but geographically distant sediments of the Northern Paleohemisphere in Russia and China (*5*). Malformations also occur in *Klausipollenites*, multitaeniate bisaccates, and nonsaccate *Ephedripites* pollen of end-Permian assemblages from offshore Norway (*9*). Similar abnormalities have been reported from distant end-Permian localities of the Southern Paleohemisphere: in South Africa (*K. schaubergeri* and *Alisporites* sp.) (*10*) and in central Gondwanan India (*Klausipollenites* sp. and *Hamiapollenites insolatus*) (*11*).

**Fig. 2:**
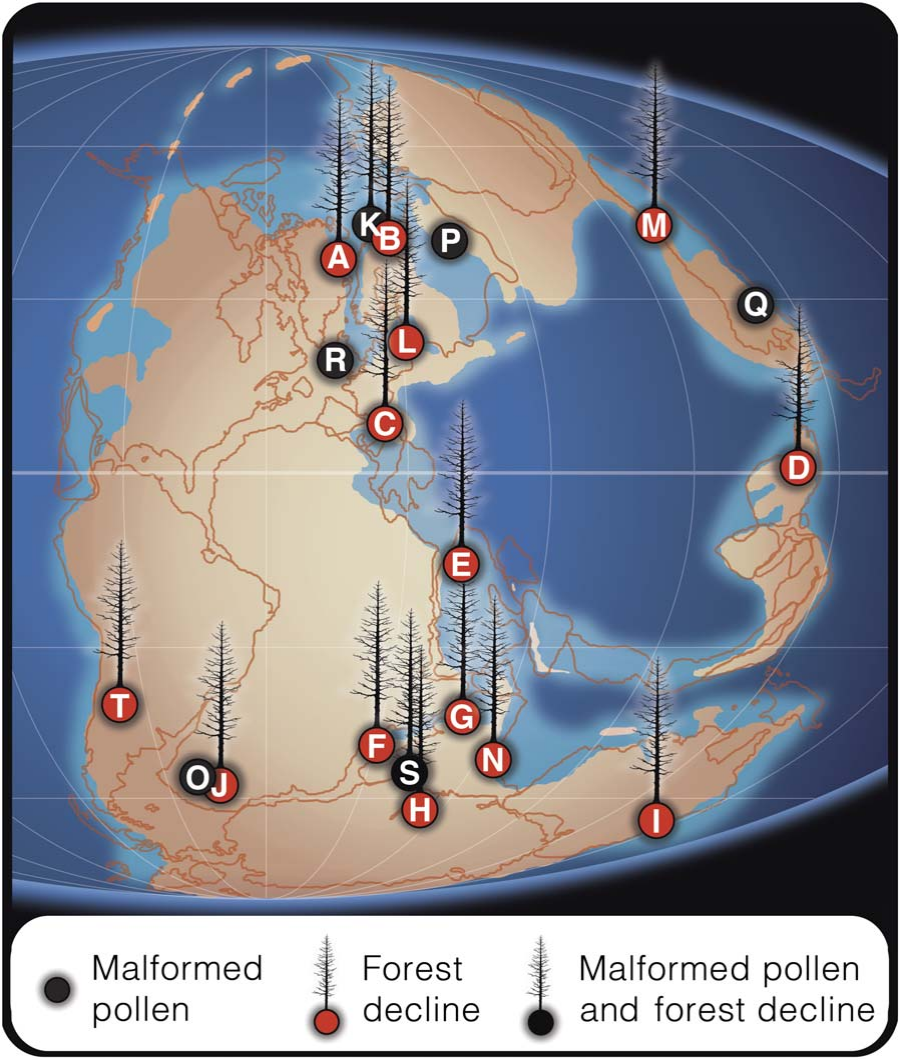
Paleogeographic distribution of Permian-Triassic palynomorph sequences featuring forest decline and/or pollen malformations. Gymnosperm turnover documented directly (**A** to **K** and **S**) and indirectly through sequences displaying gymnosperm recovery following lycopsid spore spikes (**L** to **N** and **T**). (A) East Greenland (*2*). (B) Southern Barents Sea (*66*). (C) North Italy (*26*). (D) Southwest China (*21*, *67*). (E) Israel (*23*). (F) Madagascar (*68*). (G) Pakistan (*22*, *69*). (H) Antarctica (*25*). (I) Australia (*70*). (J) South Africa (*71*). (K) Norway and central Barents Sea (*9*, *72*). (L) Western Poland (*24*). (M) North China (*73*). (N) South Tibet (*74*). (**O**) South Africa (*10*). (**P**) Nedubrovo, Russia (*5*). (**Q**) North China (*5*). (**R**) Ireland (*75*). (S) South India (*11*), and (T) Argentina (*76*). Late Permian paleogeographic reconstruction adapted from Scotese (*77*).

*Alisporites*-type grains have been found in situ within pollen organs of peltasperm and corystosperm seed ferns (*12*, *13*) and pollen cones of conifers (*14*–*16*). Multitaeniate bisaccate grains in the Northern Hemisphere are associated with late Permian peltasperms and conifers (*17*–*19*). *Ephedripites* pollen grains have been isolated from late Permian gnetalean pollen cones (*20*). *Klausipollenites* and *Hamiapollenites* were also produced by gymnosperms, but their exact botanical affinities are unknown. Some affected pollen types, such as *Alisporites* spp. (*5*, *21*–*26*) and *Klausipollenites* spp. (*5*, *22*–*27*), occur from near the paleoequator through mid and high latitudes of both hemispheres. It is therefore likely that several common, widely distributed conifer, seed fern, and gnetalean lineages were affected. These malformations were deposited amidst (and during) (*9*, *11*) forest recession episodes across the planet and massive perturbations in the global carbon cycle (Fig. 2) (*5*, *9*, *22*).

Heightened malformed pollen frequencies are hypothesized to be an indication of enhanced ultraviolet-B (UV-B) radiation (280 to 315 nm) exposure during the end-Permian (*5*). This increase in UV-B and associated radiative stress in the flora has been attributed to the pulsed release of ozone-depleting halocarbons by Siberian Traps magmatism (*5*), coeval with the crisis (*4*, *28*, *29*). Geophysical evidence supports this hypothesis—the Traps formed amidst enormous (up to 2.5 km thick) Cambrian evaporite sequences, samples of which still emit high concentrations of ozone-deteriorating methyl chloride (CH_3_Cl) and methyl bromide (CH_3_Br) in heating experiments (*29*). In addition, global atmospheric circulation models infer moderate depletion to near-total collapse of Earth’s ozone layer if metamorphic Siberian Trap emissions entered the stratosphere (*30*).

Although this hypothesis has gained support and has also been extrapolated using other lines of paleobotanical evidence (*28*), the effects of enhanced UV-B radiation on the reproductive biology of gymnosperms remain untested. Members of several living conifer genera produce pollen similar in size, proportionality, and bisaccate architecture as those of affected Permian-Triassic conifers and seed ferns, suggesting that their grains function in the same manner (*31*). Among living conifers, pines produce a phenotypically similar range of malformations to end-Permian forms (*5*). Therefore, pines provide a functionally and developmentally relevant system for evaluating UV-B influences on the reproductive biology of affected Permian-Triassic conifers, peltasperms, corystosperms, and potentially other gymnosperm lineages. Using the dwarf pine *Pinus mugo* Turra ‘Columnaris,’ we directly tested in growth chambers whether UV-B regimes modeled for the end-Permian induce pollen malformations, tree death, and/or reduced fertility. We therefore experimentally evaluated a proposed mechanistic link between Siberian Traps volcanism and end-Permian floral turnover.

## RESULTS

Five populations of *P. mugo* (*n* = 6 clones) were grown for 56 days during pollen and ovulate cone development. One population was grown outdoors under ambient UV-B conditions in Berkeley, CA, as a reference group for both the control and treatment groups in growth chambers. This outdoor tree population experienced an estimated biologically effective (BE) UV-B flux of 7.2 (kJ m^−2^ day^−1^)_BE_ (*32*). The other four populations were cultivated in growth chambers under control [no UV-B; 0 (kJ m^−2^ day^−1^)_BE_] and three enhanced UV-B treatments [54, 75, and 93 (kJ m^−2^ day^−1^)_BE_]. These treatment values correspond to modeled near-surface increases in UV-B irradiation from a late Permian background of 10 to 20 (kJ m^−2^ day^−1^)_BE_ to ozone depletion scenarios of 40 to 60 and 50 to 100 (kJ m^−2^ day^−1^)_BE_ if net emissions from the Siberian Traps and organohalogens were combined and released over 400 to <200 ky, respectively (fig. S1) (*33*). These fluxes represent maximum dosages received at the top of tree canopies. Morphological analysis was conducted on 600 pollen grains per pollen cone and on 24 pollen cones from each growth chamber population (table S3). Ovulate cone survivorship was morphologically assessed at the end of the developmental season in outdoor and growth chamber populations (table S4).

**Fig. 3:**
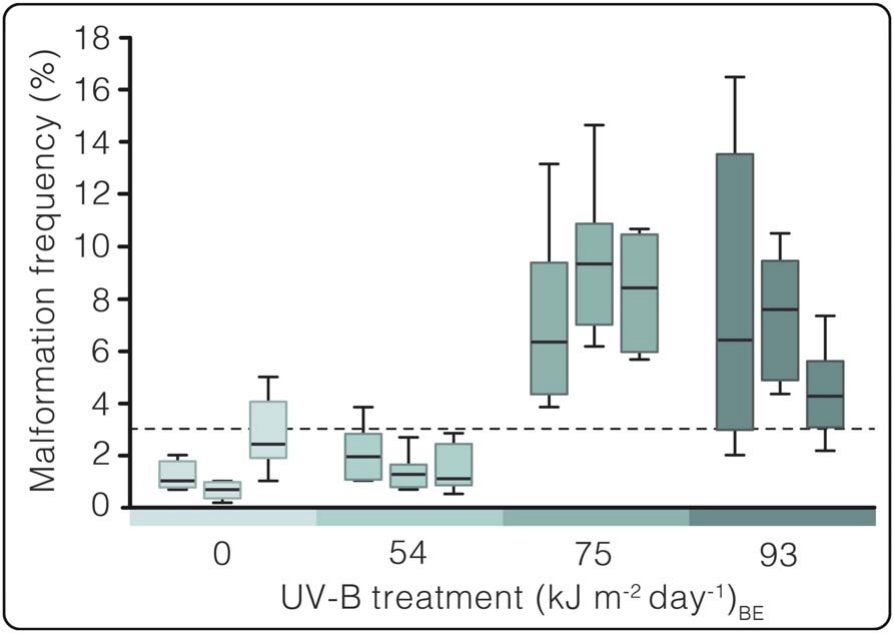
UV-B impacts on malformed pollen production in pines (*P. mugo*). Boxplots representing malformation frequencies of individual trees in control and three modeled end-Permian UV-B regimes. *Y* axis: Malformation frequency: Percent malformed grains (*n* = 600 grains per cone, eight cones per tree, three trees per treatment). *X* axis: UV-B treatment (kJ m^−2^ day^−1^)_BE_: Treatments correspond to growth chamber control (0) and end-Permian modeled (54, 75, and 93) UV-B regimes. The dashed line indicates 3% malformation frequency of stressed trees in nature (*5*). See results in table S1 and summary in table S2 for two–mixed-factor nested ANOVA.

Malformations constituted <3% of pollen yields in the control and 54 (kJ m^−2^ day^−1^)_BE_ treatments, increasing four- to fivefold in the two highest UV-B treatments (Fig. 3 and tables S1 to S3). The two highest UV-B regimes therefore induced significantly (*P* < 0.05) more pollen malformations than the control and 54 (kJ m^−2^ day^−1^)_BE_ treatments in a two–mixed-factor nested analysis of variance (ANOVA; Fig. 3 and tables S1 and S2).

**Fig. 4:**
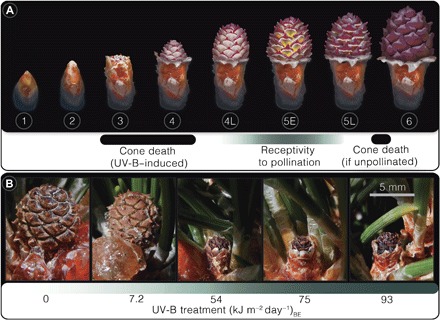
Ovulate cone developmental stages in (*P. mugo*). (**A**) Schematic of ovulate cone developmental stages in *P. mugo*. Stage descriptions: (1 to 2) Bud enclosing cone expands. (3) Cone emergence from bud scales. (4) Cone extends. (4L) Extension is completed, but ovuliferous scales (purple) are not fully opened. (5E) Ovuliferous scales perpendicular to cone axis—enabling pollen access to ovules. (5L) Ovuliferous scales swell, intercepting pollination. (6) Ovuliferous scales swollen completely, blocking pollen access to ovules (*63*). (**B**) Images of select ovulate cones from each treatment showing a typical developmental stage ~70 to 76 days following emergence. Treatments correspond to growth chamber control (0), outdoor ambient (7.2), and end-Permian modeled (54, 75, and 93) UV-B regimes. See Supplementary Text for details.

**Fig. 5:**
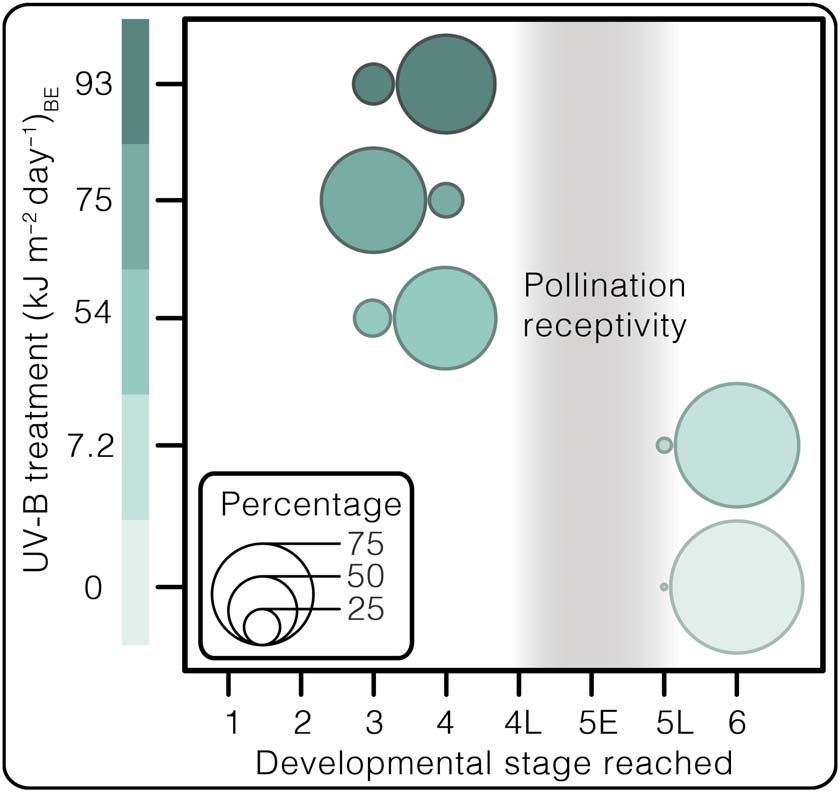
UV-B impacts on ovulate cone survivorship in pine (*P. mugo*). Bubble chart displaying *P. mugo* ‘Columnaris’ ovulate cone survivorship by displaying the percentages of cones reaching their furthest developmental stage per treatment population (*n* = 48 to 60). See table S4 for ovulate cone data.

Although all conifers survived our experiment, trees under all three enhanced UV-B regimes experienced catastrophic fertility reduction. Whereas 98 and 92% of ovulate cones produced in outdoor and control populations, respectively, successfully reached stage 6 of cone development (*n* = 60 per group), 100% of the ovulate cones died under all three heightened UV-B regimes [*n* = 48, 48, and 50 for 54, 75, and 93 (kJ m^−2^ day^−1^)_BE_ treatments, respectively; Figs. 4 and 5 and table S4]. All ovulate cones of irradiated trees died upon or just after emergence—before pollination receptivity and irrespective of canopy position or degree of self-shading (stages 3 to 4 of cone development; Figs. 4 and 5). However, the exact developmental phase and mechanism of premature ovule death requires further investigation. This period of UV-B stress did not noticeably hamper vegetative growth of *P. mugo*, nor did it permanently sterilize the trees because irradiated specimens successfully produced ovulate cones reaching developmental stage 6 (Fig. 4A) in years following the experiment outdoors.

## DISCUSSION

Previous studies of fossil and modern gymnosperm pollen suggest that yields comprising >3% malformed grains are associated with environmental stress (*5*). Here, >3% of malformation frequencies correspond to reproductive failure in conifers under heightened UV-B exposure (Fig. 3). Because ovulate cones in irradiated trees died before pollination receptivity (Figs. 4 and 5), pollen fertility was inconsequential to their fitness. Pollen germination studies were therefore not pursued.

On the basis of our results, we propose that high frequencies in phenotypic abnormalities in bisaccate pollen can be indicative of elevated UV-B exposure among other stresses and that periods with heightened abnormality frequencies in the Permian-Triassic fossil record could also have been characterized by widespread UV-B–induced fertility reduction. Under heightened UV-B, malformations are likely conservative indicators of reproductive stress because even in the 54 (kJ m^−2^ day^−1^)_BE_ treatment, all ovulate cones died prematurely, whereas trees yielded normal background malformation frequencies (1.6%; Figs. 3 and 4B and tables S1 to S3). Considering that fossilized pollen assemblages are time-averaged samples accumulated in depositional basins over many years, those yielding >3% abnormal grains could reflect either high-frequency (decadal) or prolonged (century or longer) ozone deterioration pulses. Infertility intervals corresponding to either scenario would culminate in steep fitness and population decline of gymnosperms. Intervals of volcanogenic deterioration of the stratospheric ozone layer could have triggered temporary population decline for several gymnosperm lineages rather than immediate extinction. Subsequent intervals of reduced eruption and halocarbon emissions would result in rapid ozone shield repair (within ~10 to 20 years) (*30*, *33*), allowing for gymnosperm recovery from existing populations.

Despite longevity and resiliency of individual trees, frequent or prolonged fertility reduction could diminish seed yields in regions with favorable climates and viable seed banks in those with more sporadic recruitment opportunities (for example, periodic wildfires and rare rainy seasons). Under either circumstance, progressively fewer seeds would germinate and reduced seedling populations would be subject to background mortality rates that they may not have been able to sustain. Over time, fitness reduction coupled with even modest seedling mortality could therefore have culminated in regional extirpation or even in the extinction of some Permian-Triassic gymnosperm lineages. In addition, Permian-Triassic crisis intervals appear to coincide with phases of intense greenhouse climatic conditions (*34*), during which time a concomitant fertility crisis could have severely hampered migration of some gymnosperm lineages.

Reduced fertility of several widespread and/or common gymnosperm groups could impose severe ecological consequences consistent with patterns of vegetation turnover observed in several high-resolution fossil pollen and spore assemblages [for example, figure 2 in the study of Hochuli *et al*. (*3*)]. Fluctuations in ozone column thickness may explain pulsed extirpation, recovery, and delayed extinction of some gymnosperm lineages in palynomorph assemblages spanning both hemispheres from the latest Permian to 500 ky into the Early Triassic (Fig. 2) (*3*). Concomitantly, with the pulsed vegetation turnover, U-Pb dates of Siberian Trap deposits indicate the onset of flood volcanism ~300 ± 126 ky before faunal extinctions and cessation ~500 ky into the Early Triassic (*4*). In addition, paleomagnetic studies suggest that the volcanism during this interval was likely pulsed in nature (*35*). Whereas extrusive eruptions appear concentrated into two phases, intrusive magmatism likely persisted throughout the Angaran Province during the interim, after the marine biotic crisis (*4*). Passive emissions of organohalogens could therefore have continued between eruptive phases via contact metamorphism of host sediments containing petroleum-bearing evaporites (*4*, *29*).

Volcanogenic SO_2_ aerosols would likely have contributed strongly acidic rain (*30*) in Permian-Triassic Siberia. This, together with heavy metal pollution, is another stress associated with pollen malformations in modern conifers of industrially polluted regions (*36*, *37*) and could thus have contributed to the observed changes in Permian-Triassic palynological records as well. However, Siberian Trap–induced acid rain would have been regionally confined to the Northern Hemisphere (*30*, *33*), whereas malformed pollen (*10*, *11*) and gymnosperm turnovers (*22*, *25*) also occurred in the Southern Hemisphere (Fig. 2). The extent of Siberian Trap stratospheric ozone destruction, in contrast, would have encompassed the entire planet (*30*).

Although other untested forms of environmental stress may well have contributed to localized pollen malformation production, UV-B radiation is uniquely capable of inducing globally consistent reproductive defects in terrestrial vegetation. This possibility may be further evidenced by an increase of fossilized unseparated lycophyte spore tetrads worldwide—a hypothesized floristic stress response to enhanced UV-B (*28*). Although these plants are interpreted to be environmental stress tolerators (*38*), further study is needed to evaluate the susceptibility of crown members in this lineage to heightened UV-B and stimuli conducive to spore tetrad production.

Through decreasing fertility of several common gymnosperm lineages across latitudes, pulsed volcanogenic ozone weakening could have repeatedly restructured dominance patterns in global vegetation. Widespread switchovers in the Permian-Triassic fossil record from taxonomically diverse gymnosperm-dominated assemblages to diversity-impoverished lycophyte/pteridosperm floras (*3*) constitute drastic reorganizations of the terrestrial food web base. These changes could have triggered food web collapse and extinction cascades within terrestrial animal communities without contribution of an abiotic kill mechanism. Similarly, models of probabilistic trophic networks predict that late Permian terrestrial vertebrate communities were likely prone to collapse via primary productivity disruption (*39*).

We have demonstrated that enhanced UV-B does not kill conifer trees outright but inhibits their reproduction. If Late Paleozoic putative relatives of modern conifers (for example, Volziales) as well as peltasperms, corystosperms, and gnetaleans were similarly sensitive to UV-B, then their ovules would have been considerably more vulnerable to its impacts. Unlike modern pines, which shield their ovules with heavily reinforced, overlapping bracts and ovuliferous scales, early conifers and seed ferns bore their ovules on sparsely foliated shoots (*40*–*42*) lacking structural protection from the external environment.

Furthermore, we found that ovulate cones were systemically killed or aborted throughout *P. mugo* canopies regardless of location or shading. This finding indicates that UV-B induces whole-plant sterility rather than acting upon individual exposed branches. As a result, self-shaded ovulate cones in the lower canopy of conifers also do not yield viable seeds under UV-B regimes modeled for the end-Permian. This observation would suggest that, under an ozone-weakening scenario, understory woody plants would be less likely to experience UV-B–induced fertility reduction than canopy-dominant trees. However, this protection would have been reduced as Permian-Triassic forests transitioned to more open woodlands during crisis intervals.

The fertility crisis hypothesis we propose was prompted by unanticipated results of an exploratory, reproductive study of a single-species cultivar over one growth season. Of course, constraints of our experimental system raise uncertainties when extrapolating long-term, widespread responses of several major plant lineages in the distant past. Nevertheless, using observable modern systems as proxies for historical processes is the basis of nearly all actualistic studies in paleobiology. Although some uncertainties raised by this investigation may not be resolvable, several are experimentally testable. For example, although we cannot affirm that malformation-producing Paleozoic gymnosperm lineages were vulnerable to UV-B stress on the basis of *P. mugo* alone, further investigations of additional early divergent conifer lineages and gnetophytes could determine whether UV-B–induced fertility reduction is more likely ancestral or lineage-specific within crown gymnosperms. In addition, even if heightened UV-B was a primary contributor to Permian-Triassic gymnosperm declines, it is unclear why some seed-free vascular plant lineages were not similarly affected. Experimental studies focusing on nearest living relatives of these lineages may elucidate whether UV-B could elicit contrasting responses between major plant groups.

We found that end-Permian–modeled UV-B regimes did not permanently sterilize the trees or noticeably impact their vegetative growth. It is therefore possible that *P. mugo* may have acclimatized to these conditions if exposed for subsequent years. Similarly, previous multiyear studies have documented acclimatization in vegetative growth of conifer seedlings to heightened UV-B, although under substantially lower regimes than this study—1×, 2×, and 3× ambient modern conditions (7.7 kJ m^−2^ day^−1^) (*43*). However, our results imply that conifer vegetative and reproductive sensitivity to UV-B may be decoupled. Consequently, even long-term studies on vegetative growth and ecophysiology of conifers could underestimate the consequences of ozone-weakening events on forest health and sustainability.

Several features of our study system imply that fertility reduction may not have been easily mitigated by acclimatization under a prolonged treatment. *P. mugo* is a conservative system for interpreting UV-B responses in conifers because it is an alpine-adapted species and should have exceptional foliar protection from UV-B (*44*). Furthermore, our specimens were reproductively mature trees acclimatized to nutrient-poor substrates under full sunlight outdoors preceding the experiment. Oligotrophic substrates are associated with many natural conifer habitats across the Northern Hemisphere (*45*) and can convey reduced UV-B sensitivity in plants (*46*). Moreover, somatic mutations appear to accumulate in conifers under prolonged UV-B exposure, suggesting that damage symptoms may be delayed in long-lived trees (*47*).

Considering the severity of response in stress-primed, alpine-adapted mature conifers to only one growth season of end-Permian–modeled UV-B regimes, we caution extrapolating reproductive acclimatization on the basis of previous vegetative seedling or juvenile tree studies. Although it is difficult to approximate a satisfactory cutoff for an acclimatization experiment on trees that could live centuries, further multiyear studies will be necessary to evaluate whether UV-B–induced fertility reduction is temporary or perennial.

Our results suggest that modern conifer forests may be considerably more vulnerable to anthropogenic ozone layer depletion than expected. Had the 1987 Montreal Protocol to curb anthropogenic ozone-depleting Chlorofluorocarbons not been implemented, global stratospheric models suggest a worldwide average of 67% reduction in ozone column thickness from 1980 to 2065 (*48*). This reduction would have been global in extent and year-round, with near-total destruction of the stratospheric ozone layer in the tropics and a 550% increase in DNA-damaging UV wavelengths in Northern Hemisphere midlatitudes by 2065 (*48*). Given that 34% of the world’s conifer species today are vulnerable or threatened with extinction [figure 3 in the IUCN Red List of Threatened Species assessment (*49*)], implementation of the Montreal Protocol may have prevented widespread conifer decline and, potentially, extirpations. Proposals to curtail restrictions on industrial ozone-depleting pollutant emissions should therefore be treated with extreme caution.

Our experimental approach contributes biological data challenging the broadly popularized paradigm that ecosystem turnovers associated with mass extinctions result from kill mechanisms (catastrophic mortality of organisms)—rather than termination of evolutionary lineages. These findings, together with fossilized pollen records, imply that Permian-Triassic forests would have appeared deceptively healthy at many points amidst the crisis. By analogy, the Chernobyl exclusion zone now hosts lush, park-like forests only decades after nuclear disaster (*50*). However, subtle floral and faunal malformations abound throughout the region, reflecting increased mutation rates and potentially heightened selectivity (*50*). In these cases, fingerprints of even potent selective forces on ecosystems can be highly cryptic. Therefore, environmental disasters in the past and present (for example, current anthropogenic climate change) are not always easily perceived as they unfold. Their consequences may instead manifest over generational to geologic time scales.

## MATERIALS AND METHODS

### Experimental design

In January 2013, 60 *P. mugo* ‘Columnaris’ specimens were established outdoors, fully exposed to the sun on a rooftop of the Valley Life Sciences Building, University of California, Berkeley, CA (37°52′17.2″N, 122°15′44.2″W). Trees were purchased from Iseli Nursery and grown outdoors under ambient UV-B exposure until March 2014 to minimize unusual stress responses to acute UV-B dosages during the experiment (*51*). These trees were a reproductively mature dwarf pine variant, measuring 42 to 49 cm in height, cultured in 19-cm-tall × 20.95-cm-diameter terra-cotta pots (100043015, NorCal Pottery Products Inc.). Thus, canopy apices of trees grown in pots were 61 to 68 cm in height from the ground. Terra-cotta pots were used in place of conventional plastic, and specimen labels were buried in the substrate to avoid UV-induced degradation and potential volatile release into enclosed growth chambers. Plants were grown and acclimatized to full sunlight outdoors in a predominately mineral substrate consisting of 3:2:1 pumice, sandy loam, and peat (American Soil and Stone). This substrate simulated qualities of low-nutrient sediments associated with many natural conifer habitats in the Northern Hemisphere (*45*) and served to reduce susceptibility of *P. mugo* specimens to heightened UV-B (*46*). In each pot, the substrate surface was covered by a disc of 100% opaque, biodegradable, cellulose weed barrier (WeedGuardPlus; Sunshine Paper Co.). This layer of weed barrier was used to prevent UV-B–mediated changes to below-ground microbial communities that could adversely affect plant health in confined pots (*52*). Plants outdoors were watered overhead via hose with industrial tap water (pH 8.4 to 8.9) three times weekly. Fertilizer was not supplemented to plants the year before or throughout the experimental duration to prevent artificially heightened UV-B sensitivity associated with increased substrate phosphorus and nitrogen availability (*46*, *53*).

Although all tree specimens were assigned accession numbers (PM01 to PM60) in January 2013, 30 specimens were selected for the study. These specimens were chosen because they yielded the highest numbers of pollen and ovulate cones of the group in 2013. All were outwardly robust and healthy before the experiment. Six specimens remained outdoors for baseline comparisons of ovulate cone production. The remaining 24 were transferred into growth chambers (E15, Conviron). Throughout growth chamber culture and the experiment, trees in each treatment were watered overhead with 1.6 liters of industrial tap water (pH 8.4 to 8.9) using a plastic watering can (204787CP, Fiskars) three times weekly. Photosynthetically active radiation (PAR) was administered in chambers using 16 cool white 160-W fluorescent tubes (F72T12/CW/VHO, Sylvania). Relative humidity in chambers was set to 50% for both day and night.

From 7 to 31 March 2014, plants were acclimated to 14.0°C-day and 8.0°C-night temperatures and a 12.5-hour photoperiod (approximate mean values for Berkeley, CA, for March to April 2013; table S5). Specimens were divided into four groups of six trees and redistributed into four growth chambers on 1 April 2014. In the four chambers, trees were acclimated to 16.0°C-day and 10.0°C-night temperatures and a 14-hour photoperiod (approximate mean values for Berkeley, CA, in May 2013; table S6).

One of the four chambers served as a control (no supplemental UV-B source); the other three housed two 120-V fluorescent UV-B lamps, each containing two broadband bulbs with a 312-nm emission peak and 281- to 405-nm spectral output (XX-15B, Spectronics Corporation). The emitting surfaces of these lamps were suspended 79.7 cm above each chamber floor (18.7 to 11.7 cm above tree apices) from frames constructed of bolted stainless steel L-beams. Each end-Permian treatment chamber housed one of three enhanced BE UV-B regimes [54, 75, and 93 (kJ m^−2^ day^−1^)_BE_]. These treatments correspond to 40 to 60 and 50 to 100 (kJ m^−2^ day^−1^)_BE_ UV-B fluxes inferred from modeled scenarios, in which net Siberian Trap halocarbon emissions were released over a 400- to <200-ky interval (fig. S1) (*28*). These fluxes were recorded from the height of the tallest tree specimen (68 cm above the chamber floor, 11.7 cm from the UV-B–emitting lamp surface). UV-B measurements were conducted using a UV-B sensor and a handheld radiometer calibrated before the experiment in winter 2014 (SKU 430, Apollo Display Meter, Skye Instruments Ltd.). The UV-B–emitting surface of each lamp was covered by a 0.003-mm-thick fresh sheet of clear, cellulose acetate (Grafix) to intercept UV wavelengths shorter than the spectrum reaching Earth’s surface (<~295 nm) (*54*). These filters were replaced every 7 days due to UV-induced degradation of the cellulose acetate. UV-B intensity was attenuated in the 54 and 75 (kJ m^−2^ day^−1^)_BE_ treatments by covering emitting surfaces of lamps with aluminum mesh screens (mesh size, 1.5 mm^2^; Phifer Inc.). Two sheets of mesh were stacked offset for the 54 (kJ m^−2^ day^−1^)_BE_ treatment, and one sheet was stacked for the 75 (kJ m^−2^ day^−1^)_BE_ treatment. Therefore, lamps in the 93 (kJ m^−2^ day^−1^)_BE_ treatment were only shielded by cellulose acetate.

Treatment groups were exposed to heightened UV-B regimes for 57 photoperiods (15 April to 10 June 2014). This interval encompassed pollen development and dispersal, ovulate cone development, and pollination for trees in which ovulate cones reached receptivity, stages 4L to 5L (Fig. 4A). Plants in all growth chambers received PAR for 14 hours each day. UV-B lamps emitted radiation 12 hours per day in each treatment chamber. UV-B treatments commenced 1 hour following the start of each PAR photoperiod and ceased 1 hour before darkness. This cycle simulated attenuated UV-B exposure during low-angle sunlight hours of mornings and evenings outdoors. Throughout the treatment, potted plants were manually shifted three times daily: once every 4 hours during each 12-hour UV-B photoperiod. Plants were shifted within rows of three, in the same order and direction daily. This rotation pattern ensured uniform UV-B dosage for all trees per treatment group and reduced chamber effects. Moreover, this practice guaranteed that each tree experienced acute UV-B dosage (highest under the lamp center) at the same time slot per photoperiod. Plants in the control chamber were also shifted in this manner on the same schedule for comparability. During all rotations, potted plants were shifted slowly and steadily to prevent shaking-induced ethylene stress responses (*55*). Trees were removed from all chambers after the last pollen cone was collected on 10 June 2014. Following the experiment, trees were placed back into the space where they were grown outdoors, exposed to full sunlight, and watered three times weekly.

### Applied BE dosages of UV-B

The goal for this experiment was to expose developing pollen and ovulate cones of conifers to BE UV-B fluxes in the same range as modeled for the end-Permian (*33*). To verify accuracy of the measurements, we conducted evaluations of irradiance for the UV-B lamps (Spectronics XX-15B) and spectral sensitivity of our UV-B detector (SKYE meter SKA 400 43638 + SKYE detector SKU 430) at testing facilities of Solar Light Co. (Glenside, PA). There, spectral irradiance curves were generated at four distances from our UV-B lamps, which were comparable to readings of our UV-B sensor at those same distances in growth chambers. To estimate BE UV-B flux, we multiplied the wavelength-specific fluxes (measured for 2-nm bins) by the corresponding wavelength-specific relative photon effectiveness [see figure 3 in the study of Caldwell (*56*)]. The integral of the resulting multiplied curve yielded the BE UV-B flux at a specific distance from the lamp (fig. S1). For the end-Permian, various model-reconstructed scenarios of volcanism-enhanced BE UV-B irradiation are reported, with the lowest scenario having UV-B fluxes of 30 to 60 (kJ m^−2^ day^−1^)_BE_ and the highest reaching 50 to 100 (kJ m^−2^ day^−1^)_BE_ (fig. S1) (*33*).

The tops of tree canopies in the enhanced UV-B treatment chambers (on average, 15.2 cm from the UV-B lamps) experienced UV-B fluxes of 54, 75, and 93 (kJ m^−2^ day^−1^)_BE_ for the minimum, medium, and maximum treatment levels, respectively. This range is similar to maximum values in the lowest and highest flux scenarios [that is, 60 and 100 (kJ m^−2^ day^−1^)_BE_] modeled for the end-Permian (*28*). The elevation range of pollen cones selected for this study was further in distance (between 30.2 and 37.8 cm) away from the lamps. This elevation range therefore experienced more attenuated UV-B fluxes of 26 to 31, 36 to 43, and 45 to 54 (kJ m^−2^ day^−1^)_BE_ for the minimum, medium, and maximum treatment levels, respectively (fig. S1). The range in these values roughly corresponds to the minimum values in the lowest and highest flux scenarios [that is, 30 and 50 (kJ m^−2^ day^−1^)_BE_] modeled for the end-Permian (*33*).

Although we were unable to monitor the UV-B dosage experienced by outdoor trees, they likely experienced an average flux of 7.2 (kJ m^−2^ day^−1^)_BE_ based on Caldwell-adjusted sum estimates for 300, 305, 311, 317, 325, 332, and 368 nm for the University of California, Berkeley campus (37°52’17.2"N, 122°15’44.2"W) generated by the Tropospheric UV model using data assimilated from NASA Total Ozone Mapping Spectrometer/Ozone Monitoring Instrument and Moderate Resolution Imaging Spectroradiometer satellites and National Centers for Environmental Prediction North American Regional Reanalysis surface data sets (*32*). We were also unable to supplement growth chamber control and treatment groups with enhanced UV-A (315 to 400 nm) and PAR (400 to 1700 nm) because of spatial constraints. Heightened exposure to these forms of solar radiation would also accompany ozone-weakening events. Although lower doses of UV-A radiation facilitate repair of UV-B–incurred DNA damage in pine seedlings, higher doses have been found to impose additive effects to UV-B (*57*). Therefore, although our experimental conditions deviated in some respects with ozone-weakening events in nature, the results of this study may not have been considerably altered by proportionately increased UV-A and PAR.

### Pollen cone collection

Together, 114 pollen cones (from 15 branches), 212 pollen cones (from 33 branches), 137 pollen cones (from 11 branches), and 298 pollen cones (from 34 branches) were collected from the control and 54, 75, and 93 (kJ m^−2^ day^−1^)_BE_ treatment growth chambers, respectively. The entire pollen cones produced in each tree were individually collected using stainless steel forceps just before or after opening (indicated by cone yellowing, expansion, and elongation). Pollen cones separated from the parent plant were immediately placed in plastic 1.7-ml microcentrifuge tubes (C2170, Denville Scientific Inc.) containing a solution of 95% ethanol solution [C_2_H_6_O; Chemical Abstracts Service (CAS) #64-17-5] for preservation. Cones were placed directly into ethanol rather than conventionally dried to minimize contamination, sample loss, and desiccation-induced grain distortion (*58*). Each pollen cone was accessioned with collection date, branch height, and relative degree of shading from overhanging branches (full shade, partial shade, or exposed).

Because UV-B dosage decreases with distance from the lamps (fig. S1) and malformation production rates may vary throughout a season, comparability of samples between trees and treatments was maximized by subsampling pollen collections on the basis of cone height and collection date. Specifically, we limited our comparison to cones collected in the shortest time interval within the smallest height range, yielding eight non-shaded (one exception, see note below) cones per tree, in three trees per treatment (fig. S2). This resulted in the selection of 96 cones (four treatments × three trees × eight cones per tree; table S4) that matured between 20 and 26 days after the start of the treatment (5 to 11 May 2014). These pollen cones developed on the trees between 41.9 and 49.5 cm in height from the growth chamber floor (that is, 37.8 and 30.2 cm below UV-B–emitting lamp surfaces). For corresponding UV-B dosages in this zone, see fig. S1. Note that cones sampled from specimen PM52 within this elevation range and time frame were partially shaded. Nevertheless, its cones yielded the highest mean malformation frequency in the 75 (kJ m^−2^ day^−1^)_BE_ treatment—9.5% compared to 7.1 and 8.2% for specimens PM38 and PM54, respectively (Fig. 3 and table S3).

### Pollen sample size determination

A subsample size of 600 pollen grains was used to enumerate the proportion of malformations within pollen cones harvested during the experiment. This number was based on in silico simulations (fig. S3) investigating the effect of pollen subsample size on accurately recognizing background frequencies of malformed pollen (that is, the chance of each sampled pollen grain exhibiting malformation = 0.02). This revealed that subsample sizes exceeding 600 pollen grains do not produce markedly smaller interquartile ranges relative to the increase in effort their analysis would require (fig. S3). Thus, 600 pollen grains were morphotyped from each of the 96 cones, yielding a comparison of 57,600 grains.

### Pollen processing procedure

All pollen samples were exposed to alcohol-soluble (but water-insoluble) resin acids exuded from associated cone tissues during storage in 95% ethanol solution. As a result, processing steps immersing resin-contaminated pollen in deionized water during initial processing tests resulted in clumping and adherence of grains to tube walls during centrifugation. To minimize pollen loss during decanting cycles, we developed a simplified rendition of a clearing and staining protocol for extant spores and pollen (*58*). Specifically, our protocol substitutes use of deionized water in decanting steps with 95% ethanol. Here, we provide this protocol in detail.

Microcentrifuge tubes containing individual cones and their dispersed pollen were manually shaken to encourage grain suspension. A disposable soda-lime glass Pasteur pipette and a 3-ml rubber dropper bulb (catalog nos. 13-678-6A and 03-448-26, Fisherbrand) were used to extract and transfer a small subsample of pollen-containing ethanol solution to empty microcentrifuge tubes. The transferred samples were topped with 95% ethanol solution, manually shaken, and centrifuged at 3800 rpm for 30 s in a microcentrifuge (5415 D, Eppendorf). The ethanol solution was removed using the same pipette, and the tube was filled to 75% volume with 10% potassium hydroxide solution (KOH; CAS #1310-58-3) and placed into wells of a 110-W dry block heater (12621-104, VWR International) set to 130.8°C for 6 min (on high heat, with both low and high dials set to “7”). The temperature was measured from the bottom center of wells by a K-type thermocouple sensor probe attached to a liquid crystal display instant-read digital thermometer (HYELEC MS6501). During the KOH hot bath, tubes were removed from wells with a toothpick every 45 s, uncapped to release pressure, manually shaken, and placed back in the well. This 10% KOH cooking step served to hydrolyze cellulose and lyse protoplasts, leaving the exine of pollen grains (that is, the sporopollenin outer layer retained in fossilized palynomorphs) intact but transparent (*58*).

Following heating, the tubes were vented and recapped to relieve pressure, manually shaken, centrifuged, decanted, and filled with 95% ethanol solution. After three further centrifuge/decanting cycles with 95% ethanol solution, two drops of safranin O dye (C_20_H_19_N_4_Cl; CAS #447-73-6) were added, and the tube was topped with 95% ethanol solution, manually shaken, and centrifuged. Eighty-five percent to 90% volume of the dye solution was then decanted via pipette, leaving behind a solution highly concentrated in dyed pollen. One drop of glycerol (C_3_H_8_O_3_; CAS #56-81-5) was added and then mixed with the sample using a flat toothpick (Diamond, Jarden Corp.).

Small pollen subsamples were extracted using a glass pipette/dropper bulb and transferred to the centers of standard microscope slides on a slide warmer set to 56°C (XH-2002, Premiere, C&A Scientific Co. Inc.). Pollen solution subsamples were left exposed on the slide warmer in a fume hood for approximately 5 min to evaporate residual ethanol and ensure that the residue contained predominately stained pollen and glycerol. On each slide, the pollen grain–containing solution was gently mixed with one small (diameter, 2 to 3 mm) melted cube of glycerin jelly (7.63% gelatin, 53.4% glycerin, and 0.76% phenol) using a flat toothpick and then enclosed by a 22 mm × 30 mm × 0.16 mm cover glass (12-544-A, Fisherbrand). Mounted slides were left for 30 min on the slide warmer (set to 56°C) to encourage spread of sample/medium under the cover glass. Slides remained in a fume hood at ~22°C for 2 days after mounting to off-gas residual water and ethanol vapor from under the cover glass. Cover glasses were then sealed by either paraffin embedding or clear nail polish. Five to eight slides were prepared from each pollen cone so that 600 grains were counted.

### Pollen microscopy analyses

Pollen grains were morphotyped using a Leica DM2500 microscope fitted with a differential interference contrast system (to render contrast in transparent grains) using a 20× objective lens (506503, Leica Microsystems Inc.). All grains were morphotyped by one individual (J.P.B.) to minimize deviations in protocol. Data pertaining to treatment group, cone elevation, and shading were omitted from all sample slide labels to ensure blind comparison. Malformed grains were categorized into subgroups using a 40× objective lens (506144, Leica Microsystems Inc.). Grains bearing two sacci were counted as phenotypically normal, whereas those having more or less than two sacci were scored as malformations (Fig. 1, J to P, and table S3). Normal and malformed grains were tallied using a two-key desktop laboratory counter (Clay Adams, Becton, Dickinson and Co.). Malformation categories were tallied using a 24-key desktop laboratory counter (Vary Tally, Veeder-Root Inc.). Exemplary normal and malformed grains were imaged through the view of a Plan Apo 63× oil objective lens (506187, Leica Microsystems Inc.) for the highest degree of Apochromatic and flat field correction. Grains were imaged with a Nikon Digital Sight DS-Fi1 camera with live feed into a Nikon DS-L2 control unit (Nikon Corp.). Extended depth-of-field images were generated using CombineZP (*59*). Images were compiled as jpegs (2560 × 1920 pixels, 300 dots per inch).

### UV-B impacts on whole-plant reproduction

Whole-plant fitness across treatments was assessed by morphological assessment of in vivo ovulate cone survivorship throughout canopies of all growth chamber and outdoor specimens. We used stage 6 (Fig. 4A) as a proxy for pollination success rather than in vitro pollen germination because successful pollination was required to reach this stage (*60*) and even pollen with lethally irradiated nuclei in some plants can germinate, produce pollen tubes, and penetrate ovules in vitro (*61*). For each ovulate cone, accession number and elevation were written on a 1.5 cm × 1.0 cm label cut from water-repellent paper (978-1-932149-89-0, Rite in the Rain, JL Darling LLC) using a no. 2 pencil. These labels were threaded through stainless steel 38 mm × 0.25 mm insect pins (#000, Ento Sphinx) taped to pine needles in fascicles adjacent to each ovulate cone using label tape (L-3000-3, GeneMate, VWR International).

We categorized ovulate cone survivorship using developmental stages described for controlled pollination of *Pinus* by the U.S. Department of Agriculture (*62*–*64*) with subdivisions of stages 4 and 5 (Fig. 4A) (*60*, *65*). Ovulate cones emerged from buds between 20 and 26 April (stage 3; Fig. 4A). During this time, pollen grains were found adhered to exposed surfaces throughout each growth chamber. Pollen was therefore available in all growth chambers to ovulate cones reaching the stages of receptivity (4L to 5L; Fig. 4A). Survivorship of all ovulate cones was morphologically assessed on the basis of photographs taken of each cone ~70 to 76 days after emergence (5 to 11 July 2014) with scale. By this point, ovules pollinated with successfully germinating pollen had reached stage 6 (Fig. 4A) (*60*). Cones that appeared unpollinated expanded until nearly completing stage 5L (Fig. 4A) (*60*), when their ovuliferous scales ceased growth and hardened as the cone desiccated.

## Supplementary Material

http://advances.sciencemag.org/cgi/content/full/4/2/e1700618/DC1

## SUPPLEMENTARY MATERIALS

Supplementary material for this article is available at http://advances.sciencemag.org/cgi/content/full/4/2/e1700618/DC1 Supplementary Text fig. S1. BE UV-B fluxes versus distance from UV-B lamps in growth chamber experiments. fig. S2. Pollen cone subsampling strategy. fig. S3. Sample size and accuracy of malformed pollen frequency determination. table S1. Results of a two–mixed-factor nested ANOVA of malformed pollen frequencies. table S2. Summary of a pairwise, two–mixed-factor nested ANOVA of malformed pollen frequencies. table S3. Pollen malformation frequencies, percentages, and index per tree. table S4. Ovulate cone survivorship across treatments. table S5. Temperature settings during growth chamber experiment. table S6. Photoperiod settings during growth chamber experiment. References (*78*–*80*)
