# Supplementary material for: UV-B–induced forest sterility: Implications of ozone shield failure in Earth’s largest extinction
Source: Sci Adv. 2018 Feb 7;4(2):e1700618. doi: 10.1126/sciadv.1700618 (PMC5810612; doi:10.1126/sciadv.1700618)
Supplement: http://advances.sciencemag.org/cgi/content/full/4/2/e1700618/DC1 [file supp_4_2_e1700618__index.html]

Science Advances | Science Advances

## Supplementary Materials

**This PDF file includes:**

- Supplementary Text
- fig. S1. BE UV-B fluxes versus distance from UV-B lamps in growth chamber experiments.
- fig. S2. Pollen cone subsampling strategy.
- fig. S3. Sample size and accuracy of malformed pollen frequency determination.
- table S1. Results of a two–mixed-factor nested ANOVA of malformed pollen frequencies.
- table S2. Summary of a pairwise, two–mixed-factor nested ANOVA of malformed pollen frequencies.
- table S3. Pollen malformation frequencies, percentages, and index per tree.
- table S4. Ovulate cone survivorship across treatments.
- table S5. Temperature settings during growth chamber experiment.
- table S6. Photoperiod settings during growth chamber experiment.
- References (*78–80*)

Download PDF

**Files in this Data Supplement:**

- Adobe PDF - 1700618\_SM.pdf
