# Supplementary material for: UV-B–induced forest sterility: Implications of ozone shield failure in Earth’s largest extinction
Source: Sci Adv. 2018 Feb 7;4(2):e1700618. doi: 10.1126/sciadv.1700618 (PMC5810612; doi:10.1126/sciadv.1700618)
Supplement: http://advances.sciencemag.org/cgi/content/full/4/2/e1700618/DC1 [file 1700618_SM.pdf]

Science Advances

AAAS

advances.sciencemag.org/cgi/content/full/4/2/e1700618/DC1

Supplementary Materials for

UV-B–induced forest sterility: Implications of ozone shield failure in Earth's largest extinction

Jeffrey P. Benca, Ivo A. P. Duijnstee, Cindy V. Looy

Published 7 February 2018, *Sci. Adv.* **4**, e1700618 (2018)
DOI: 10.1126/sciadv.1700618

This PDF file includes:

- Supplementary Text
- fig. S1. BE UV-B fluxes versus distance from UV-B lamps in growth chamber experiments.
- fig. S2. Pollen cone subsampling strategy.
- fig. S3. Sample size and accuracy of malformed pollen frequency determination.
- table S1. Results of a two–mixed-factor nested ANOVA of malformed pollen frequencies.
- table S2. Summary of a pairwise, two–mixed-factor nested ANOVA of malformed pollen frequencies.
- table S3. Pollen malformation frequencies, percentages, and index per tree.
- table S4. Ovulate cone survivorship across treatments.
- table S5. Temperature settings during growth chamber experiment.
- table S6. Photoperiod settings during growth chamber experiment.
- References (*78*–*80*)

Supplementary Text

Fig. 1.

(**A**–**H**) Accession notation: *Sample-slide; England finder coordinate*. (**A**) 6403373-8; H35. (**B**) 6403369-8; W40. (**C**) 6403361. (**D**) 6403373-8; L34. (**E**) 6403400-2; T18/3. (**F**) 6403400-12; N24/3. (**G**) 6403381-11; Y24/2. (**H**) 4748/36; N33. Specimens with accession number starting with 640– are housed at Geoscience Australia while 4748– are housed at the Palaeontological Institute, Moscow. Fossilized pollen images courtesy of S. Afonin. Grains published: (**A**, **E**–**G**) Fig. 4 in (*5*). (**H**) (*78*). (**I**–**P**) Notation: *Specimen-slide; England finder coordinate, treatment*. (**I**) 200778-200505; T45-3, 93 (kJ m-2d-1)$_{BE}$. (**J**) 200779-200684; P47-3, 54 (kJ m-2d-1)$_{BE}$. (**K**) 200780-200715; L47, 75 (kJ m-2d-1)$_{BE}$. (**L**) 200781-200746; P45, 75 (kJ m-2d-1)$_{BE}$. (**M**) 200782-200714; L45-2, 75 (kJ m-2d-1)$_{BE}$. (**N**) 200783-200743; M40, 75 (kJ m-2d-1)$_{BE}$. (**O**) 200784-200661; N42-4, 54 (kJ m-2d-1)$_{BE}$. (**P**) 200785-200568; N44, 54 (kJ m-2d-1)$_{BE}$. *P. mugo* pollen was collected from cones under accession 200281. Extended depth of field (EDF) images were generated using CombineZP (*55*) by J.P.B.

Fig. 3.

Boxplots represent individual tree specimen pollen yields. Tree specimens in the following order–left to right. Notation: species-specimen number. Species: *Pinus mugo* (PM). (Control [0 (kJ m^{-2}d^{-1})$_{BE}$]): PM03, PM17, PM24. (54 (kJ m^{-2}d^{-1})$_{BE}$): PM15, PM36, PM44. (75 (kJ m^{-2}d^{-1})$_{BE}$): PM38, PM52, PM54. (93 (kJ m^{-2}d^{-1})$_{BE}$): PM10, PM28, PM44. Lower and upper limits of the boxes correspond with first and third quartiles (based on interpolation), the horizontal line in each box represents the median, and whiskers give

minimum and maximum values in the data. See results in table S1 and summary in table S2 for two-mixed-factor nested ANOVA.

Fig. 4, A.

Stages compiled based on controlled pollination studies of *Pinus* (*62–64*) amended with substages of 4 and 5 (*60*, *65*). Digitally vectored illustrations rendered by J.P.B. using Adobe Illustrator® CS6 (San Jose, CA, US).

Extended list of acknowledgements

J.P.B. thanks T. Dawson, A. Doban and D. Apodaca for growth chamber support, as well as R. Kirchholtes, S. Kauppinen, W. Hsiung and C. Williams for assisting in plant maintenance during travels.

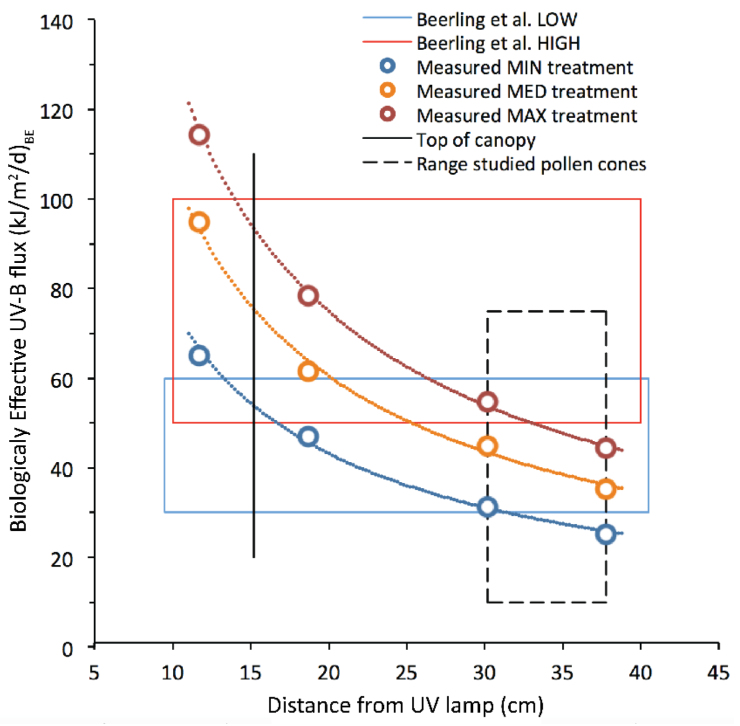

fig. S1. BE UV-B fluxes versus distance from UV-B lamps in growth chamber experiments. MIN, MED and MAX refer to the three treatment levels of enhanced UV-B irradiation. Red and blue boxes indicate the range of fluxes in the highest and lowest irradiation scenarios modeled for the end-Permian (*33*). The vertical black line indicates the position at the average top of tree canopies while the black dashed box represents the distance range from the lamp from which pollen cones were analyzed. Open circles give the measured BE UV-B flux values, dotted curves represent the modeled relation between BE UV-B flux and distance ($y = 485\ x^{-0.807}$, $y = 678\ x^{-0.807}$ and $y = 840\ x^{-0.807}$ for MIN, MED and MAX, respectively).

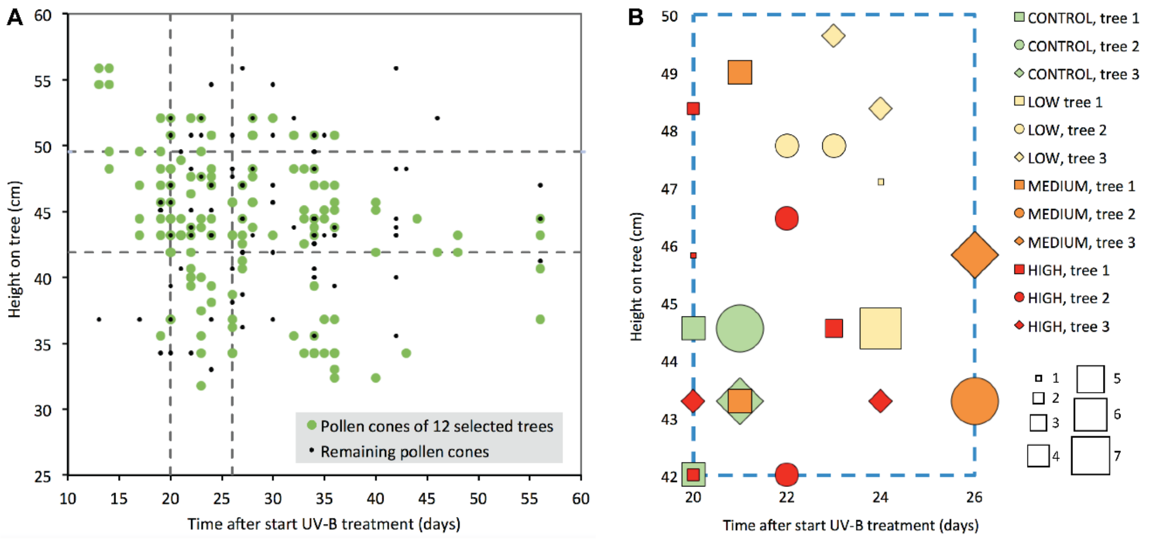

fig. S2. Pollen cone subsampling strategy. (**A**) Collection timing and canopy height of the 634 pollen cones that developed during the experimental period. Several cones were harvested on the same day and from the same height on the tree resulting in overlapping data points. (B) Collection timing and canopy height of the 96 pollen cones selected for further study. The symbol size refers to the number of cones of a particular tree harvested on the same day and canopy height.

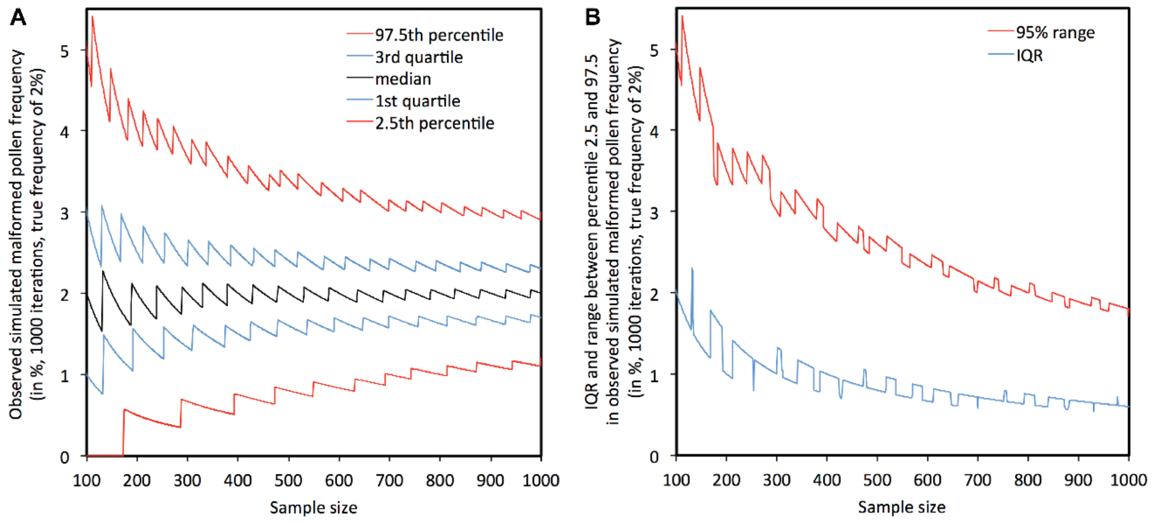

fig. S3. Sample size and accuracy of malformed pollen frequency determination. During *in silico* simulations we simulated pollen subsamples of various sizes (100–1000 grains), in which each grain had 2% chance of being malformed. Each of the 901 different sample sizes was replicated 1000 times. (**A**) Frequency summary of observed malformation frequencies in simulated pollen subsamples. Various percentiles of observed simulated pollen malformation frequencies versus sample size are indicated. (**B**) Difference between the first and third quartile (interquartile range, or IQR) and between the 2.5th and 97.5th percentile (95% range) versus sample size.

table S1. Results of a two–mixed-factor nested ANOVA of malformed pollen frequencies. Significance of treatment and tree-specific effects were tested. Listed are sum of squares (*SS*), degrees of freedom (*df*), mean sum of squares (*MS*), *F*-statistic (*F*), and *p*-value. Data consisted of 4 treatments, with 3 trees each, 8 cones per tree and 600 pollen grains per cone analyzed for malformations. In the analysis, treatment type was treated as a fixed factor; which tree within each treatment was treated as random factor.

	SS	*df*	*MS*	*F*	*p*-value
Treatment (fixed factor)	30979	3	10326	22.301	0.00031
Tree; nested in treatments (random factor)	3704	8	463.04	2.3617	0.02414
Within	16469	84	196.06		
Total	51152	95	538.45		

table S2. Summary of a pairwise, two–mixed-factor nested ANOVA of malformed pollen frequencies. The *F*-statistic is shown in the supra-diagonal part of both sub-tables; *p*-values in the sub-diagonal parts. Data consisted of 4 treatments, with 3 trees each, 8 cones per tree, and 600 pollen grains per cone analyzed for malformations. In the analysis, treatment type was treated as a fixed factor; which tree within each treatment was treated as random factor. Bold values refer to either significant differences between pairs of treatments, or significant differences between the trees within pairs of treatments. Numbers in the labels of enhanced UV-B treatments refer to the dosage of biologically effective UV-B at the top of the canopy, in (kJ $m^{-2}d^{-1})_{BE}$.

	Treatment (fixed factor)				Tree; nested in treatments (random factor)			
	Control 0	UV-B 54	UV-B 75	UV-B 93	Control 0	UV-B 54	UV-B 75	UV-B 93
Control 0		0.016	**51.158**	**16.155**		**7.187**	**2.602**	**2.829**
UV-B 54	0.9063		**81.503**	**19.922**	**0.0002**		1.590	**2.217**
UV-B 75	**0.0020**	**0.0008**		1.619	**0.0495**	0.1947		2.011
UV-B 93	**0.0159**	**0.0111**	0.2722		**0.0364**	**0.0835**	0.1104	

table S3. Pollen malformation frequencies, percentages, and index per tree.

Percentages rounded to the nearest tenth.

Chamber treatment (kJ $m^{-2}d^{-1})_{BE}$	Tree	Pollen surveyed (n)	Σ Malformation frequency	% Malformed	Malformation index*	Malformation classes				
						0 Sacci	1 Saccus	3 Sacci	4 Sacci	≥ 5 Sacci
Control [0]	PM03	4800	58	1.2	0.8	0	0	49	7	2
Control [0]	PM17	4800	30	0.6	0.4	3	0	22	5	0
Control [0]	PM23	4800	131	2.7	1.8	1	0	116	12	2
54	PM15	4800	98	2.0	1.3	5	0	90	2	1
54	PM36	4800	64	1.3	0.9	3	0	58	3	0
54	PM44	4800	69	1.4	0.9	11	0	50	4	4
75	PM38	4800	339	7.1	4.6	4	0	328	5	2
75	PM52	4800	456	9.5	6.3	0	0	438	16	2
75	PM54	4800	395	8.2	5.4	0	1	371	22	1
93	PM10	4800	384	8.0	5.3	3	0	371	10	0
93	PM28	4800	354	7.4	4.9	4	0	334	14	2
93	PM42	4800	213	4.4	2.9	2	0	205	6	0

table S4. Ovulate cone survivorship across treatments. Survivorship indicated by cones reaching stage 6. See (Fig. 4, A) for developmental stage descriptions, and (Fig. 4, B) for images of representative cones from each treatment. Percentages rounded to the nearest tenth.

Stage reached	Treatments (kJ $m^{-2}d^{-1})_{BE}$									
	Control [0]		Outdoor [7.2]		54		75		93	
	Count	%	Count	%	Count	%	Count	%	Count	%
1	-	-	-	-	-	-	-	-	-	-
2	-	-	-	-	-	-	-	-	-	-
3	-	-	-	-	12	25	37	77.1	14	28
4	-	-	-	-	36	75	11	22.9	36	72
4L	-	-	-	-	-	-	-	-	-	-
5E	-	-	-	-	-	-	-	-	-	-
5L	1	1.7	5	8.3	-	-	-	-	-	-
6	59	98.3	55	91.7	-	-	-	-	-	-
Σ no. ovulate cones	60		60		48		48		50	

table S5. Temperature settings during growth chamber experiment. Temperatures based on U.S. Climate Data for Berkeley, CA, US (*79*). Temperatures rounded to the nearest tenth.

Interval, 2013	Average high (˚C) T_{HIGH}	Average low (˚C) T_{LOW}	Mean (˚C) T_{AVE}	Chamber day setting (˚C) T_{AVE}	Chamber night setting (˚C) T_{LOW}
March–April	20.0	7.9	14.0	14.0	8.0
May	22.7	10.1	16.4	16.0	10.0

table S6. Photoperiod settings during growth chamber experiment. Photoperiods based on estimations of sunrise and sunset times for Berkeley, CA, US (*80*). Values listed in hours : minutes. Chamber photoperiod rounded to the nearest half hour.

Interval, 2013	Estimated photoperiod (first day of interval)	Estimated photoperiod (last day of interval)	Averaged photoperiod	Chamber photoperiod
March/April pooled	11:23	13:45	12:37	12:30
May	13:47	14:36	14:12	14:00
